# A Linked List-Based Algorithm for Blob Detection on Embedded Vision-Based Sensors

**DOI:** 10.3390/s16060782

**Published:** 2016-05-28

**Authors:** Ricardo Acevedo-Avila, Miguel Gonzalez-Mendoza, Andres Garcia-Garcia

**Affiliations:** Department of Postgraduate Studies, Tecnológico de Monterrey, Campus Estado de México, Atizapán de Zaragoza, Estado de México 52926, Mexico; mgonza@itesm.mx (M.G.-M.); garcia.andres@itesm.mx (A.G.-G.)

**Keywords:** embedded computer vision, field programmable gate array (FPGA), object detection

## Abstract

Blob detection is a common task in vision-based applications. Most existing algorithms are aimed at execution on general purpose computers; while very few can be adapted to the computing restrictions present in embedded platforms. This paper focuses on the design of an algorithm capable of real-time blob detection that minimizes system memory consumption. The proposed algorithm detects objects in one image scan; it is based on a linked-list data structure tree used to label blobs depending on their shape and node information. An example application showing the results of a blob detection co-processor has been built on a low-powered field programmable gate array hardware as a step towards developing a smart video surveillance system. The detection method is intended for general purpose application. As such, several test cases focused on character recognition are also examined. The results obtained present a fair trade-off between accuracy and memory requirements; and prove the validity of the proposed approach for real-time implementation on resource-constrained computing platforms.

## 1. Introduction

Consumption of automated image recognition technology has been growing steadily over the past few years [1,2,3]. Today, the use of embedded camera-equipped devices is common and is often found in commercial hardware ranging from laptop computers, mobile phones and personal digital assistants (PDAs) to smart vehicles and automated surveillance systems. Modern image processing applications must support complex computations on large streams of visual data. This technology could be provided by personal computers; however, power consumption, size and mobility are commonly desired and often forbid the use of such devices in many vision applications. Dedicated image processing platforms offer a combination of embedded computing power and video sensors that present the resources needed to perform real-time image processing operations with a focus on efficiency maximization (*i.e.*, performance and manufacturing cost).

Embedded vision (EV) is a subset of computer vision focused on systems where sensors are often encapsulated by the device they control. Processing is performed on-board, and system design constantly requires a trade-off between computationally-intensive algorithms and resource consumption. EV applications are diverse, and implementation ranges from entertainment interaction systems, such as video games [4] and virtual reality systems [5,6], to human assistance tools, like robots and other autonomous devices [7]. In the automotive industry, embedded sensors-based systems are commonly focused on challenging tasks like pedestrian detection [8], lane departure prevention [9,10] or obstacle detection [11]. In the smart surveillance field, computer vision is used as a video analysis accelerator. Examples include vehicle traffic monitoring [12], event surveillance and sophisticated biometrics analysis of human features (e.g., face or fingerprints) [13].

Common EV algorithms include image enhancement, image segmentation, pattern recognition and object detection. Object detection deals with the extraction and measurement of the objects that are present in the scene. This task can be further sub-divided into two operations: background segmentation and blob analysis. Background segmentation involves the separation of background and foreground information, while blob analysis focuses on the identification of specific foreground regions that represent object shapes. The first step of blob analysis is blob detection. Blob detection is used to detect connectivity among image objects (*i.e.*, adjacent pixel areas sharing one common color value). In typical EV operations, bottlenecks arise due to high data transfers (image information stored on memory devices), algorithm implementation (serial *vs.* parallel) and hardware resources (CPU *vs.* GPU). One possible solution to build up an efficient EV system is to develop custom hardware dedicated to process raw image information.

Nonetheless, only a small sub-set of software-based algorithms are suitable for implementation on embedded hardware, and few solutions have been developed for resource-limited devices [14,15,16,17]. This article is focused on the design of a one-scan low-memory blob detection algorithm. The proposed algorithm analyzes the input image as it is buffered from the image acquisition stage. Only relevant information for blob identification is stored. Detection is based on the fast management of linked-lists data structures to process blob information based on object shape. We propose the use of a data tree intended for classification of each blob according to its detection history. This makes analysis direct, as each class involves a defined set of operations that do not require broad computational overhead.

As a proof of concept, the algorithm is evaluated in a case study consisting of an EV system realized on an Altera Cyclone III EP3C120F780I7 (119K Logic Elements) field programmable gate array (FPGA) development board as part of a mobile object recognition system intended for smart surveillance analytics. We effectively minimize memory consumption while achieving real-time processing. The FPGA implementation is capable of analyzing video graphics array images in three formats: Quarter-VGA (320 × 240), VGA (640 × 480) and Super-VGA (800 × 600). The blob detection subsystem consumes a total of 665 logic elements. Processing rates vary from 221 FPS (QVGA) to 88 FPS (SVGA).

The remainder of this paper is organized as follows: Section 2 presents related concepts and work. Section 3 discusses the proposed algorithm for blob detection. Section 4 examines an example application built on FPGA hardware. In Section 5, we present and evaluate the results obtained. Finally, in Section 6, conclusions are drawn.

## 2. Related Concepts and Work

The object detection stage typically receives a binary image as input. A binary image denotes foreground pixels with a color value of 1, while the the background pixels are denoted with 0. This image is also referred to as the foreground mask and is produced by a background segmentation technique that can range from a simple thresholding operation (*i.e.*, the use of a fixed rule to determine background and foreground pixels) to an elaborate statistical-based algorithm (*i.e.*, the use of historic models to determine the class of a new pixel) [18,19].

Region connectivity is the main criteria used to identify object shapes in a foreground mask. Connectivity in a pixel area is described as the spatial proximity between pixels in a binary image [20]; each connected area is often called a binary large object (blob) [21]. In order to isolate each blob as one entity, a labeling process is carried out to partition the input image into connected components. A labeled blob has a unique identifier that can be used to further measure general properties, like shape, position and state [22]. Blob processing algorithms are typically raster scan-based. A raster scanned image is displayed as a horizontal sequence of pixels starting in the upper left-hand corner of the image gradually moving from left to right, top to bottom. Scan-based blob detection techniques are classified into two main categories [23,24,25]: recursive [26] and sequential [27].

Recursive approaches assume that read-accesses to the input image are unlimited. The method is simple, but inefficient in resource-limited machines. The main issue being the number of image scans over the input depends on the complexity of the image. Recent techniques have been developed to optimize recursive-based algorithms. The authors in [28] present a method featuring a label connection table that stores assigned labels throughout the input image; it substantially reduces execution time by performing forward and backward image scanning. This feature allows one to resolve duplicated or conflicting labels.

Conversely, sequential methods typically require two image scans and can be optimized to process more than one image row at a time. This technique is used when there is limited storage. An example of this approach is presented by [29]; where the authors propose a union-find structure-based algorithm that re-uses temporary labels. This scheme allows representing labels and their relationship as data trees, where labels with few children are absorbed by labels with a bigger number of descendants.

A simplified case of sequential methods are one-pass only algorithms. The main idea of the algorithms in this sub-category is to detect and label blobs in just one image scan. In order to achieve this, [30] proposes the implementation of boundary object detection. This technique focuses on estimating blob contours and filling their interior with a label that depends on neighboring pixel areas.

Examples of blob detection algorithms based on these ideas are well documented. The implementation typically targets general computing platforms. The work in [31] presents a fast connected component labeling software. The application is built using the Open Source Computer Vision (OpenCV) framework. The algorithm explores the input image in search of connected sub-regions via pixel neighborhoods; several sub-regions are then integrated into a single region. The approach uses a partial neighborhood mask that leaves small unconnected holes. Nevertheless, the trade-off substantially reduces labeling computation time. The software runs at 30 FPS on a PC under Windows 7 with a single core Intel Atom N280 CPU at 1.6 GHz and 1 GB of memory. Image acquisition is performed by a web camera; image sizes range from 176 × 144 to 1280 × 1024.

The authors in [32] propose a real-time blob detection algorithm for software video surveillance. Their method relies on applying a correction phase before component detection is carried out. The correction applies morphological filtering (*i.e.*, filling up image holes) while labeling is being performed. The technique is called neighbor foreground pixel propagation (NPP); which effectively removes small un-connected pixel regions. The software runs on a Windows PC with a 3-GHz Pentium 4 Core CPU and 3 GB of main memory; the frame resolution is 320 × 240. Although the algorithm seems to achieve high-speed processing, maximum throughput is not reported.

A blob detection algorithm for digital documents is proposed by [33]. Their goal is to detect glyphs on different kinds of images. Again, frame sub-regions are exploited to accelerate processing. The authors benefit from the fact that text documents have less foreground information than background. The algorithm analyzes the image looking for foreground objects; once an object is found, its position is stored in a vector. This position is used to assign a label in a second image scan. The software processes frame sizes of 850 × 1600, 2500 × 3500 and 800 × 1200; it runs on a Windows XP Intel 2.2 GHz Core 2 Duo PC with 3 GB RAM. Maximum size images (2500 × 3500) are processed in 132 ms. General frame rates for each image size are not reported.

The work in [34] proposes a general Laplacian of Gaussian (gLoG) filter for detecting elliptical blobs in medical images. The algorithm also computes blob features, such as center, scale and orientation. The approach works by first applying a Gaussian filter to reduce image noise and then using a Laplacian operator to detect edges. The algorithm is implemented in MATLAB and is not intended for real-time execution. It runs on a Windows PC with a 2.4-GHz CPU and 4 GB of memory. It processes 800 × 600 images at a rate of 3.5 min/image (0.0047 FPS).

GPU-based solutions are often examined in the image processing literature. However, due to their high power consumption, they are rarely used as embedded computing platforms. The authors in [35] develop a full face detection system implemented on a NVIDIA GTX 470 graphics card using CUDA. The system carries out several image processing tasks, but focuses on blob detection using a parallel integral image computation. This operation is optimized to compute integral images in parallel employing a multiscan kernel operation. Next in the processing pipeline is a parallel Haar evaluation filter that analyzes the input image for face-like features. The system analyzes HD images of a size of 1920 × 1080 at 35 FPS. The parallel integral image is computed in 2.3 ms.

Applications involving blob detection for embedded platforms have been increasing in recent years. One example is presented in [36]; where the authors suggest a face recognition algorithm implemented with a complementary metal-oxide semiconductor (CMOS) sensor housed in parallel with a digital signal processor (DSP). The set-up is intended for image analysis acceleration. The algorithm is executed in 4.2 ms on 640 × 480 (VGA) images at 30 FPS and detects the faces of five persons. In [37], the authors minimize the computational overhead for embedded blob detection with the proposal of a parallel algorithm. The input image is partitioned and distributed to four concurrent processing elements (PE). Each PE compares the labels of an input pixel and four adjacent neighbors. The corresponding label is output based on this information, requiring at least four image scans. A merging phase delivers the final labeled image.

The approach presented in [38] proposes an algorithm based on a method called pseudo partitioning. This technique allows labeling without the use of a merging phase. The algorithm runs on an array of nine DSPs. Each DSP performs component labeling for a portion of the image, based on neighborhood search. This solution does not require DSP intercommunication, yielding good performance and speed. An algorithm called light speed labeling (LSL) is introduced in [39]. This blob detection method is designed for the implementation on RISC processor architectures. It is focused on CPU pipeline optimization by the reduction of conditional evaluations and memory accesses. It is based on a concept called line-relative labeling, a segment-based adjacency detection method that is used to simplify equivalence between image rows. The algorithm requires three image scans for corrected input to be generated.

A custom computer platform approach for vehicle tracking and surveillance is suggested by [40]. According to the authors, the hardware architecture has been chosen to support fast prototype development, flexibility and performance. Image acquisition is achieved by an embedded monochrome CMOS image sensor. This device delivers images with a VGA resolution at 30 FPS. The processing unit is an array of Texas Instruments (TMS320C64x) DSPs. The DSP array performs the actual image processing algorithms with a power of 80 billion instructions per second. Custom embedded architectures can also be designed in order to achieve real-time image processing. FPGA-based implementations have gained focus in computer vision due to their capabilities of developing software, as well as custom hardware [41,42,43], boosting performance. FPGA technology offers the possibility to develop a full system on a chip (SOC); where custom processors can analyze data coming directly from the acquisition stage.

The FPGA architecture presented in [44] is based on the two-pass algorithm introduced by [45]. The algorithm operates on a binary image. In the first image scan, background pixels are filtered out and foreground pixels are classified based on their neighborhood labels. A second pass is used to resolve possible connectivity problems. The hardware implementation is achieved on a Spartan-3A DSP FPGA board, working at 27 MHz and processing 640 × 480 (VGA) images at 60 FPS. Another implementation is found in [46]; the authors employ a single-pass component-labeling algorithm. Their system is equipped with a custom Shack-Hartmann wavefront sensor (SHWVFS) connected to a charge-coupled device (CCD) array, from which 224 × 244 frames are acquired. The detection algorithm is implemented on a Xilinx Spartan-6 150LX FPGA working at 70 MHz. The SHWVFS device allows a high-speed throughput of 905 FPS.

The authors in [47] propose a technique based on the identification of intersecting pixels on each image row and column. The image is first pre-processed by a chain of filters (e.g., grayscale conversion, median filtering and a final threshold operation). Pre-processing is intended to boost detection results; a series of logical tests are then carried out to label each intersecting pixel. Their solution is designed for visual navigation systems and also computes object area and centroid. The algorithm is realized on a Xilinx Virtex V FPGA board, operating at 100 MHz. Their system processes 100 × 100 images at 4545.45 FPS (0.22 ms) and 1024 × 1024 images at 61.72 FPS (16.2 ms). It detects a total of five objects and utilizes four blocks of RAM.

The work by [48] exploits parallel processing inherent in hardware design by partitioning the input frame into vertical slices. The partitioning idea is similar to the work presented in [37]. Each image slice is analyzed for connected components concurrently by a pixel processing component. A central unit merges all label data from separated pixel processing components. The algorithm is implemented on a Xilinx Virtex 6 XC6VLX240T FPGA clocked at 136.4 MHz for 1024 × 1024 input frames. The architecture is optimized for high throughput, achieving a frame rate of 1.1 GPixels per second (1049 FPS for a 1024 × 1024 image).

An updated architecture by the same authors is presented in [49]. The new features include detection of image patterns aimed to minimize label errors, a control structure used to detect the last pixel of an image, optimizing memory resources, recycling of previous labels and simplification of the labeling processes by reducing the number of label lookups per pixel. The hardware architecture detects blobs in images ranging from 640 × 480 to 7680 × 4330. The implementation is evaluated using different FPGA families, including Xilinx Virtex 6, Spartan 6 and Kintex 7 boards. Evaluation on Kintex 7 yields a working frequency of 170.3 MHz, using 548,000 bits of block ram memory (BRAM) and processing images of 256 × 256 up to 7680 × 4320 ultra-high definition (UHD8k) frames. A worst-case throughput of 136.42 MPixel/s is reported.

The authors in [50] propose minimum memory consumption by implementing a one-scan algorithm. Based on pseudo-partitioning; image sub-regions are processed in search of connected components. The solution operates on binary images and propagates labels as soon as a connected component is detected. Small sub-region images allows label information to be stored in only 75.6 kbits of on-chip RAM. The architecture can process frame sizes of 512 × 512, 1280 × 720, 1024 × 1024, 1920 × 1280 and 1280 × 1280. The blob detection system is realized on a Stratix II FPGA working at 97.4 MHz; it achieves a frame rate of 49 FPS while processing a 1280 × 1280 frame.

The authors in [51] propose a blob detection system intended for virtual reality (VR) applications. The system detects blobs and computes their center points in real time. Blobs are identified by running an adjacency test using a four- and eight-pixel neighborhood. After a blob has proven adjacency, its coordinates are computer based on bounding boxes. It works by searching the minimal and maximal XY coordinates of each blob. Blob attributes are then written to a FIFO structure. The system is realized on a DE2 Altera FPGA board running at 125 MHz and processing 640 × 480 frame sizes at a maximum of 50 FPS, without monitor output. Resource consumption is of 13,311 logic elements and 239,316 bits of memory.

One of the main challenges of designing an object detection algorithm for embedded hardware is balancing the trade-off between memory throughput, processing time and detection accuracy [52]. It is possible to partially solve these issues with the implementation of data structures used to represent blob information over the input image [24,53,54,55]. A label can be propagated from a parent node throughout the rest of the tree if contiguity has been proven; saving further processing time. It is possible to use linked-list to represent blob information. A linked-list is a node structure that contains a pointer to a preceding or successive node [56]. This list can quickly update its root node without affecting its child nodes, offering design flexibility while supporting dynamic data.

In this article, we present an algorithm for blob detection based on linked-lists for embedded implementation. It prioritizes minimum data storage and fast processing. The algorithm is examined and is used in a prototype blob detection system deployed on FPGA hardware. FPGA technology enables reconfigurable hardware development on the fly and can be also used to develop complete pipelined systems in one physical chip. The blob detection architecture is also integrated with a blob tracker sub-system. Results obtained while tracking blobs in a video surveillance application are presented, as we compare the specifications of similar architectures studied in the literature. The algorithm is also evaluated for general purpose detection within a character recognition scenario. This is a particularly challenging area of blob detection, as blob geometry is often complex, nevertheless, it helps us evaluate the algorithm under the most extreme conditions.

## 3. Blob Recognition Process

### 3.1. Overview

The algorithm receives a binary foreground mask in which all detectable objects are rendered in white. The mask is transferred from the video source throughout the application pipeline as a continuous stream of pixels. Each pixel is received from left to right and top to bottom, in a raster-graphics format. Pixel connectivity along the input image is determined on a two-row basis. This technique exploits the fact that connectivity is present if two rows of the same color share a horizontal coordinate. The binary nature of the input image allows the use of a data compression method aimed to reduce the volume of processed information, as each row can be reconstructed in sequences of smaller numerical intervals (e.g., run length encoding (RLE)). The algorithm runs the compressed data through several tests aimed to classify each blob in a particular detection case where a unique label is assigned. Each case performs a series of operations based on the object shape and previous detection information. Once classified, each blob is assigned a corresponding label. The label can be a non-repeating integer, but usually takes the form of a color value, with each connected blob represented by a different color.

Blob information is stored in data structures called bins. Each bin contains the label and the current pixels linked with a detected object in the input image. Bin management is fast, but not easy; it involves managing data from multiple list data structures. Each list is updated differently according to established detection rules. Simple shaped blobs (e.g., circles and rectangles) can be detected in a single pass with almost no further problems. However, complex shapes that are first detected as separated blobs and later found to be joined (e.g., concave objects) will need an extra correction step.

**Figure 1:**
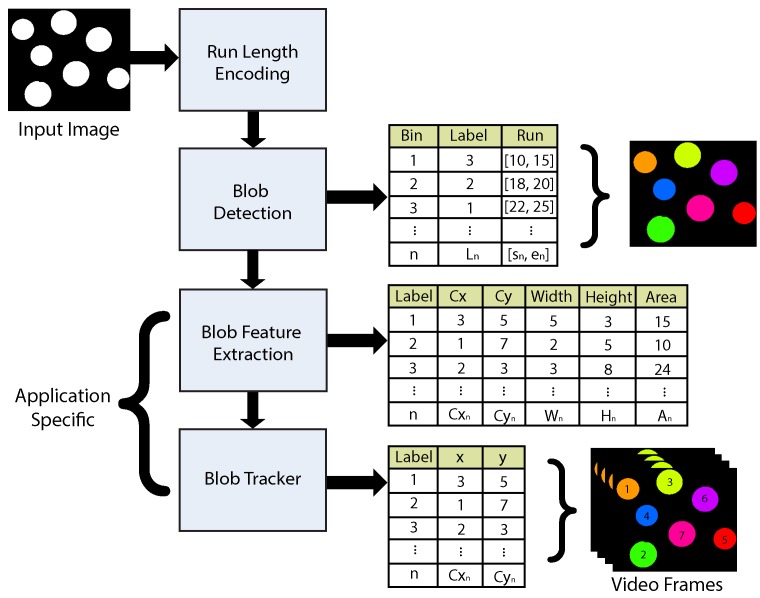
Blob detection and tracking. Full system overview.

The correction phase relies directly on the information contained in the linked-lists. Correction is achieved by merging blobs with common bin information and using a single label for the complete shape. After each blob has been identified and labeled as a group of connected pixels, feature properties can be computed. Feature extraction is usually application dependent. In this work, the blob detection algorithm performance is evaluated with a simple tracker for object motion. The tracker uses a minimum distance vector (MDV) obtained for different blob metrics, such as centroid and area. This information allows identifying each detected blob trough time regardless of its location on the scene, as long as their geometrical properties remain reasonably consistent. Figure 1 depicts the full overview of a blob detection and tracking system.

### 3.2. Connectivity Test

**Figure 2:**
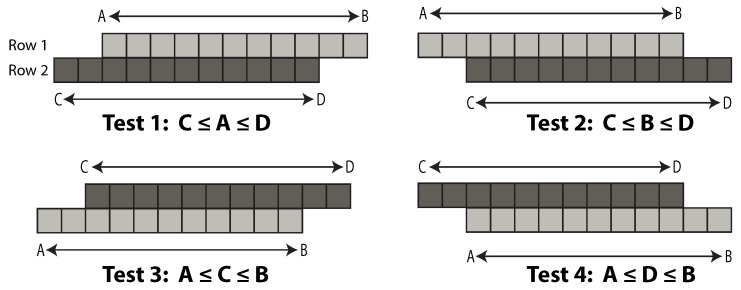
The four tests that comprise the row connectivity test.

The proposed approach involves analyzing two image rows (*runs*) per processing step. The lower row is considered part of the upper row only when both share a common horizontal point. In such a case, the rows belong to a parent blob and a single label must be assigned. The test of connected (*i.e.*, adjacent) runs is not trivial, however, as each upper row run (n) has to be tested with each lower rower run (m). This a computational problem of complexity n×m. In the worst case scenario n=m, and complexity increases to $n^{2}$. The connectivity test is composed of four logical comparisons. Consider Figure 2; each run is described by a starting and ending value. Two rows are overlapped if one of the four possible tests is valid (*true*) for two given runs with starting values *A* or *C* and ending values *B* or *D*.

### 3.3. Bin Data System

Once a run is detected, it must be linked with either a new or existing label. A new label will be assigned only if the connectivity test fails in a single processing step. A *bin* data structure is used to efficiently keep track of each blob and their associated information. Bins can store object and label data using a minimum amount of resources (e.g., an integer word to encode the current label used). A bin can easily be implemented using an array or a list.

A set of three main list structures is used through this algorithm. A list called bin list exclusively contains all of the detected blobs and their assigned bins. A second list, called label list, is used to store labels. A third and final list structure, the free bins, is employed to check which bins are currently under use and which are free. The latter is also useful for recycling unused bins and keeping control of correct bin assignments. The maximum number of detectable objects will be directly related to the maximum number of bins (*i.e.*, total entries in the list) supported by the computing platform.

### 3.4. Data Structure Dependencies

**Figure 3:**
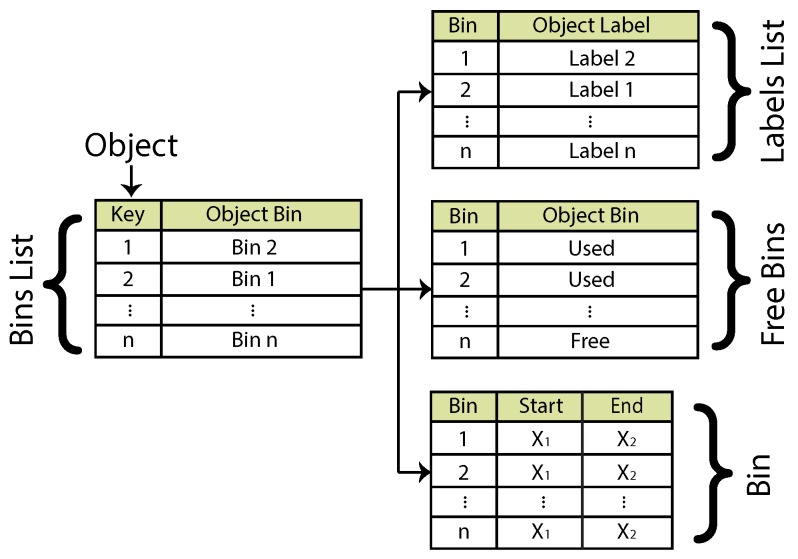
Relationships between linked-lists and bin data structure.

Each data structure can be thought of as a linked-list. Data structure linking depends on the current blob portion that is being processed; a partially-detected blob is referred to as an object. The current object generates a node key according to its position. Keys are implemented as integers that increment as subsequent objects are encountered. A pair of rows is processed from left to right. The initial key assignment is, therefore, dependent on the object’s initial position in the image. The object key is used to link the object to a bin; the bin is then linked to a label. The list of current free and used bins is also linked to the tree structure. An extra data structure can be used to store the start and end points of a detected object. Figure 3 depicts the linked-lists used and their interdependence.

### 3.5. Detection Cases Classification

The typical operation of the three linked-lists is as follows: An object is detected, and a unique key is generated. A new label is provided only if the object is not part of an existing blob. If the object is part of a previously detected blob, its key is linked to an existing root node. Same bin information (*i.e.*, root label) is used for all of the objects that are part of a common parent blob. The approach is direct; however, several complex scenarios can be encountered depending on the shape and parent information of an object. In this section, some basic detection cases are examined, and the use of linked-lists is exploited to obtain a successful detection under most circumstances. For the next part, assume a two-row processing from left to right is performed at all times, unless otherwise stated.

#### 3.5.1. Detection Order Changes (Bin ≠ Object Order)

**Figure 4:**
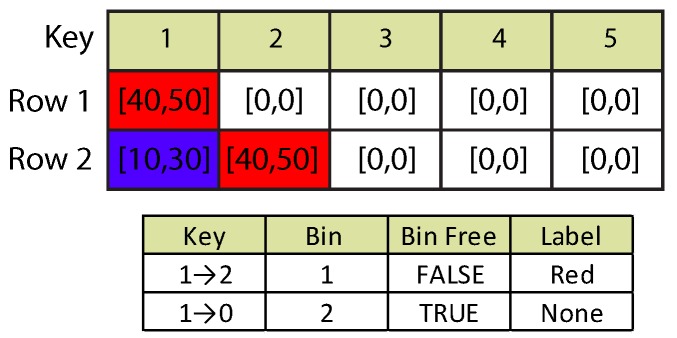
Detection Case 1: detection order changes.

Suppose a new object begins detection at one frame. A new bin and label are assigned. However, midway through processing, a second object located below the original object is detected for the first time. The new object displaces the original object as first in the object detection stack. This is an instance of the case where detection order changes and the assigned bin is different from object order. Refer to Figure 4. The first data array represents the white pixel lines in compressed RLE format. Each cell has been labeled with a different color to illustrate overlapping runs. Row 1 has one pixel line (labeled in red) as the currently-detected object.

In Row 2, a second object (labeled in blue) is detected before Object 1. The algorithm adjusts the linked-lists structures by updating the objects keys accordingly. The second data array presents the adjusted lists after processing of both rows is completed. The key of the red object has been updated from one to two, while its linked bin, bin status and label value present no further change. Meanwhile, the blue object shows no adjacency with any other run; thus, it is not yet assigned a label or a bin. Selection of a new bin depends on the information available on the free bins list.

#### 3.5.2. Long Run

A long run is encountered when disjointed objects (*i.e.*, multiple runs in a row) are part of a parent blob previously detected. This situation is typically found when processing concave-down-like shapes. In this case, the connectivity test will be valid for more than one run. Under this scenario, objects contained in different bins will share the same label.

**Figure 5:**
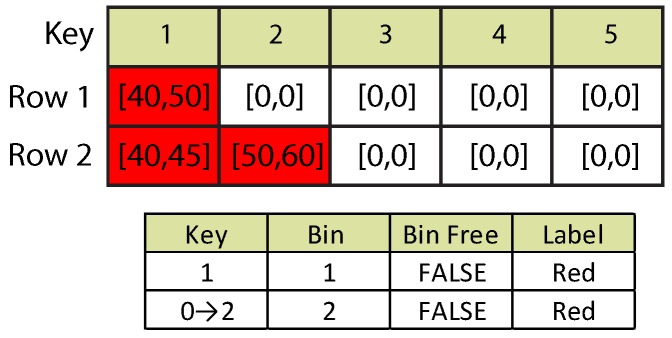
Detection Case 2: long run.

Figure 5 depicts the situation. Notice that two different runs can share a parent blob. Both runs in Row 2 have proven adjacency with the run in Row 1. For Run 2, a new key and bin have been issued using the information of the original parent blob. The successive presence of multiple long runs can affect the original detection order of the objects, as a parent blob can spawn several child objects (*i.e.*, a blob can be forked in other objects). This also means that an object can be inserted between two originally disjointed objects. To deal with such a case, the number of displaced positions produced by each child object must be accumulated before re-adjusting final object keys.

A variation of the long run case is the reverse long run. It is presented when disjointed objects are first detected in a row and then are found to be joined by a parent blob in the next processing step. A common instance of this case is found when a concave-up blob is processed. This case can be solved by merging all object labels. However, if the objects have not been found to share a parent blob until the last moment, a final correction must be applied.

#### 3.5.3. Blob Termination

**Figure 6:**
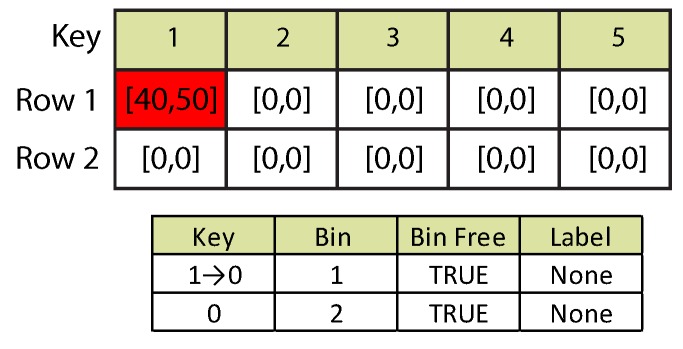
Detection Case 3: blob termination.

Whenever a previously-detected blob reaches an end (e.g., is no longer detected in the following row), its data structures must be removed. Blob termination is straightforward. The last object that is part of a blob will not share adjacency with any other run computed afterwards. This situation is the cue for the object’s bin and label to be discarded. The free bins list will also update the current status for that bin, marking it available for future use. Figure 6 shows the blob termination case.

### 3.6. The Bin-Based Blob Detection Algorithm

**Figure 7:**
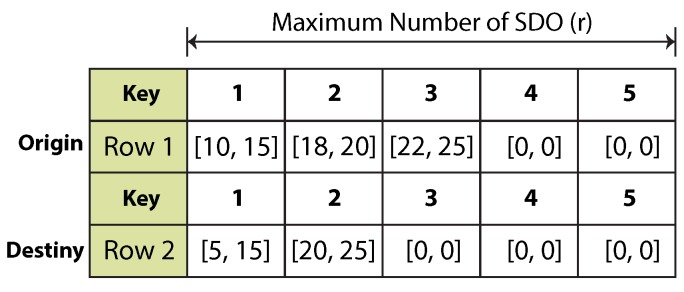
Run list. The first row is called the origin row, while the second is the destiny row.

Once the components required by the blob recognition algorithm have been explained, we can proceed to outline the full approach for general blob recognition. The algorithm’s input is two binary image rows encoded in RLE format. Each row contains a variable number of encoded white pixel lines (runs). After the foreground mask information is encoded in runs, a principal run list is generated. This list is depicted in Figure 7. The number of columns represents the maximum number of simultaneously-detectable objects (SDO) the algorithm is able to process. This value is denoted by the constant *r*. As mentioned in Section 3.2, processing is of n×m complexity, where *n* is the number of runs contained in the first row and *m* the number of runs in the second row; in both cases, n≤r and m≤r.

**Figure 8:**
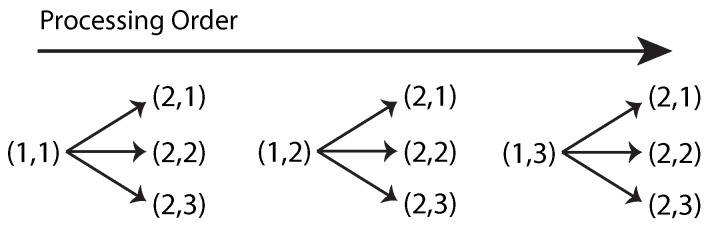
Sequential processing of two runs in a list of SDO=3. Each node is depicted as (Row,Key).

Bin and label management is progressive and depends on chronological operations, which means each encoded run needs to be processed sequentially. Each origin element in the run list is processed with each destiny element. Once all elements in the destiny row are traversed, the origin key is incremented in one unit. After the origin key reaches its maximum value, processing for both rows is completed. Figure 8 shows the sequential order in which two runs are processed. The following step is to perform the connectivity test. The test involves four logical comparisons. A logical function, called the adjacency function (Equation (1)), summarizes the overall result of each individual test.

(1) $$\begin{array}{c} AdjFun\Rightarrow \{(c\leq a)\wedge (a\leq d)\}\vee \{(c\leq b)\wedge (b\leq d)\}\\ \vee \{(a\leq c)\wedge (c\leq b)\}\vee \{(a\leq d)\wedge (d\leq b)\} \end{array}$$

Algorithm 1 shows the general outline of the blob detection algorithm. The inputs required are the three linked lists, as well as the lists of run length encoded image rows. Consider that processing is performed on two image rows at a time, top to bottom. Once two adjacent runs are detected, one of the following two cases may occur: both runs share the same position in the run list or one of both runs is spread across a row. In the former case, run keys share the same value. However, in the latter case, run keys are different. If the current destiny run shares its key and position with an origin run, an existing parent bin and label might have been already assigned. The algorithm requests a new bin using the origin key as the index value in the bin list. If the requested bin has a value of zero, the current bin is not under use. The algorithm assigns a new bin using the information available in the free bins list. Label assignation follows using the current bin as the index. As before, a value of zero denotes an unassigned label.

If the positions of two detected bins are not the same, two sub-cases can be presented depending on the occurrence of a previous detection. The first sub-case involves the long run situation described previously. In this situation, the algorithm updates the object’s origin key while maintaining its bin unaffected. Each object detected as part of a long run must also have its label merged with its parent label. The second sub-case must be evaluated for two possible situations. If a new object proved adjacent to a previous run, a reverse long run might occur. If this is not the case, the final situation must involve a previously-detected run that just changed position.

**Algorithm 1.** The blob detection algorithm.
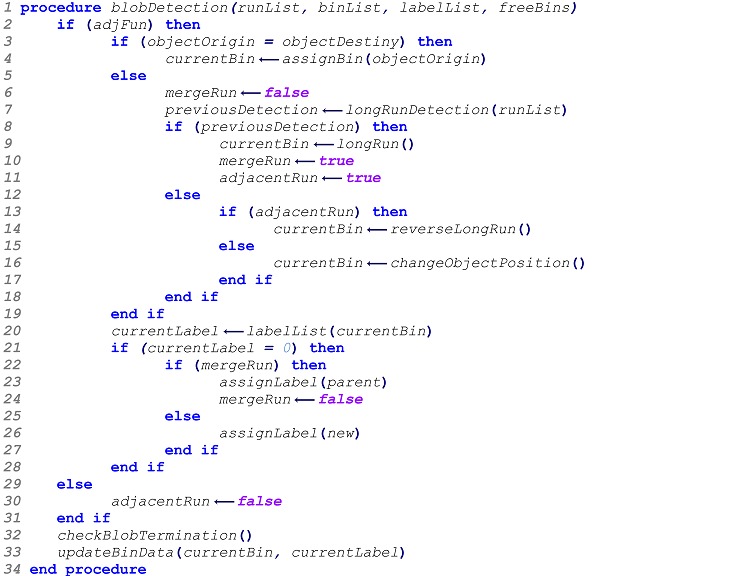


The next step is to detect a blob’s end. The algorithm cannot detect the termination of an object unless all of the elements of both rows have been processed. Every time a valid run fails the adjacency test, a flag is set, and its bin and label are marked. The flag is unset if adjacency is eventually found. Finally, bin, label and coordinates are updated. Parent initial propagation is also handled during the update procedure.

### 3.7. Label Correction (Bin Merging)

The goal of the blob recognition algorithm is to detect blobs using the minimum possible amount of memory while maintaining real-time processing speed. The two-row approach serves both purposes; however, its biggest weakness is the correction of objects that turn out to be connected at the very last minute (*i.e.*, concave shapes). To address this issue, a label correction stage has been implemented. It operates on bin data instead of raw-image information.

**Figure 9:**
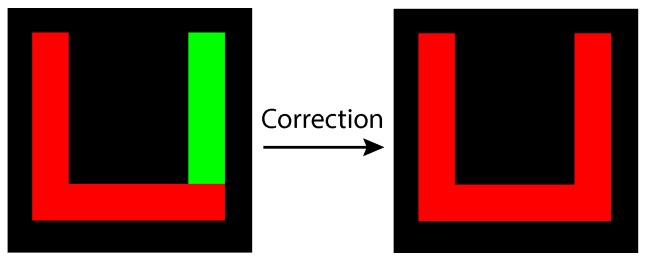
Concave up shape before and after correction.

Its principle is very similar to the connectivity test. If two different bins need to be merged, they must share a common point (*i.e.*, a common run). The approach involves traversing through all of the bins in current use in search for common run points. If a common point is found, the label of the first object is copied to the second object; all remaining data structures are left unaffected. The correction phase is active every time an image scan is finished and does not require an additional pass. Nonetheless, this operation introduces extra latency to the overall system pipeline. Figure 9 illustrates an input blob after correction is applied.

## 4. Application Example

We have evaluated the results of the proposed blob detection approach in an automated surveillance context, as part of an embedded vision-based architecture that has been implemented on an FPGA. The blob detection architecture has been described and documented entirely in VHDL, a very strict, but powerful low-level hardware description language (HDL). VHDL offers early and fast simulation at the register-transfer logic (RTL) and gate level, close-to-implementation synthesis and tool/technological independence. Inter-module portions of the full automated surveillance have been also described using Verilog.

**Figure 10:**
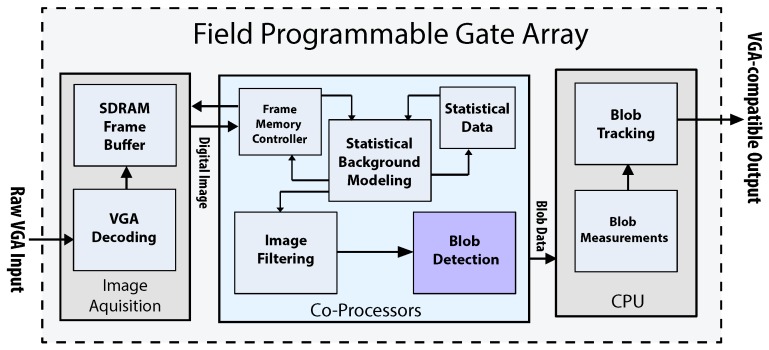
Application example: complete video surveillance embedded system.

In the image acquisition stage, the system receives raw input provided by a video source (e.g., video camera or DVR). Raw image data are typically received as a Bayer-filter pattern and then converted to RGB pixels. The RGB information is then formatted into VGA-compatible frames. Input frames are buffered into SDRAM sequentially (e.g., $pixel_{\left(0,0\right)}$ is at memory offset zero, $pixel_{\left(1,0\right)}$ is at offset one.). An additional memory controller requests pixels from SDRAM and feeds them to a background classifier component. The background classifier uses statistical data to filter out objects that do not belong to the original scene. The final binary mask is then processed by the blob detection co-processor. The component realizes the algorithm described in Section 3.6. Detection results can also be displayed on a VGA-compatible monitor. Figure 10 depicts a block diagram of the complete surveillance embedded system.

Finally, a simple software-based motion tracker module is used to estimate and register the position of the detected objects throughout time. The tracker’s main function is to resolve spatial occlusion [57,58] between two or more different shapes. Spatial occlusion causes two or more blobs to change position within two frames, thus changing the initial label assignation. Blob tracking techniques are plentiful and range from histogram-based correlation [59], HSV-based filtering [60] and blob counting [61] to more elaborated approaches, such as random sample consensus (RANSAC) [62], Markov model-based tracking [63] and Kalman filtering [64] for motion prediction.

In this paper, minimum vector distance is used as a tracking algorithm due to the simplicity and effectiveness in application [65]. This technique exploits temporal coherence, which asserts that the state (e.g., position and shape) of detected objects does not change significantly between successive time steps. The MDV is computed as the positional Euclidean distance between an origin blob and a target blob. The target blob that produces the smallest MDV is chosen as the origin blob displaced between two different frames. This technique serves as a proof of concept that validates our blob detection algorithm as a basis for higher-level operations, such as blob tracking.

**Figure 11:**
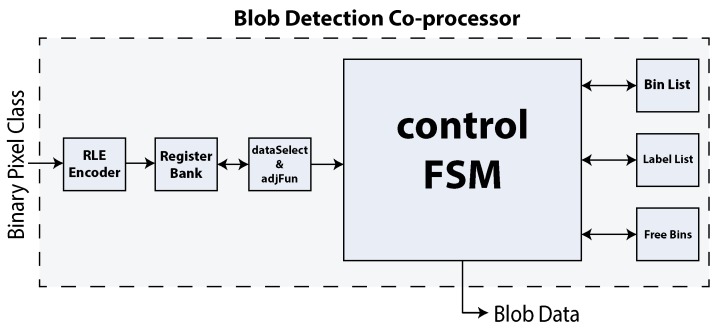
General overview of the blob detection co-processor.

A general overview of the blob detection hardware architecture is depicted in Figure 11. Data input is received as a stream of black and white pixels coming from the previous processing co-processor (*i.e.*, the background pixel classifier). Pixels are compressed in run length-based packets. Pixel packets are then stored in a register array named the register bank. This component stores a maximum of two horizontal image lines (now described as runs) and provides a constant flow of data to the core processing unit through the *dataSelect* block. Connectivity tests are then carried by a gate-based implementation of Equation (1) within the same module.

The core processing unit is the control finite state machine (FSM) block. This component implements the logic needed to realize the blob detection algorithm depicted in Algorithm 1. It relies on the three different slave register-based arrays discussed in Section 3.4. The first register array is the bin list. This structure stores part of a detected object in a specified bin. The label list is used to store a label associated with a bin. Finally, the free bins component keeps tracks of the bins that are both used and free.

**Figure 12:**
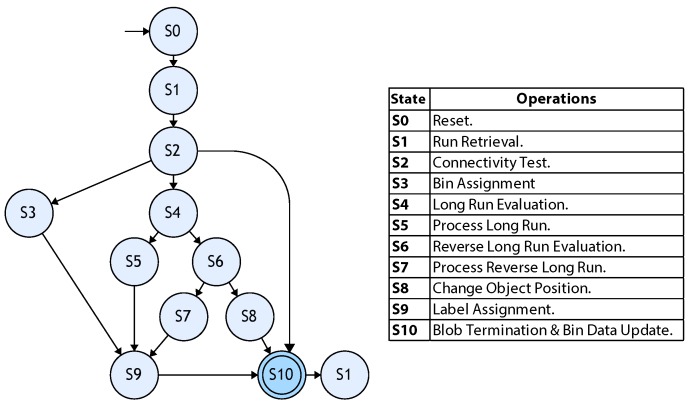
Simplified control FSM diagram for hardware implementation.

Figure 12 depicts a simplified state diagram for the hardware implementation of the control FSM. The diagram closely follows Algorithm 1. The figure does not consider latency introduced by data transfers. In the *S0* state, all signals are set to their initial values. In *S1*, a pair of new runs (pixel packets) are retrieved from the register bank. The connectivity test is performed on these runs in *S2*. Depending on the results, state flow can be directed to three different states. If the connectivity test is true and object keys are the same, control flow is directed towards *S3*. If the connectivity test is true and object keys are different, control flow is directed to *S4*. If the connectivity test fails, a consecutive detection flag (adjacent run) is unset, and the FSM goes to *S10*.

Bin assignment is performed in *S3*. *S4* evaluates the conditions for the long run case detection discussed in Section 3.5.2. If the conditions are true, the FSM goes to *S5*. If a long run is not detected, flow is resumed towards *S6*. The long run case is processed in *S5*, and the adjacent run flag is set. The reverse long run is detected using the adjacent run flag once *S6* is reached. If the flag is set, the reverse long run is processed in *S7*. If no reverse long run is detected, control flow is sent to *S8*, where the change object position operation is handled. In *S9*, label assignment is performed. The final state for one machine cycle is *S10*, where the conditions for blob termination are evaluated and all bin data are updated. The control FSM loops again to *S1* as long as image input is received.

Blob processing is unique and depends on the blob detection case currently evaluated. Some cases involve quicker operations than others. This makes overall latency difficult to calculate. Nevertheless, we can estimate the maximum latency required by the control operations in the slowest execution paths. Equation (2) summarizes the expression used to estimate the maximum latency of the control FSM during one machine cycle.

(2) $$\begin{array}{c} latency=10r^{2}-7r+11 \end{array}$$

**Figure 13:**
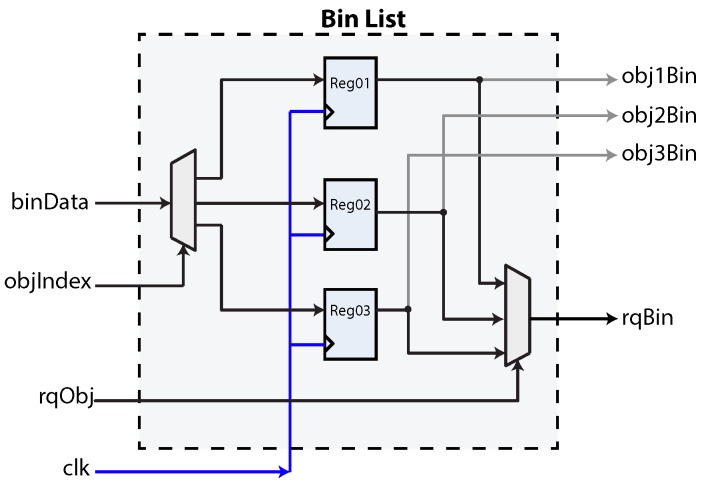
The bin list implemented as a register-based array. In this figure, a maximum of three objects can be stored.

Figure 13 shows the block diagram of a hardware linked-list implementation. The bin list structure is used to link a bin to a detected object. The data structure resembles a table, where an entry is assigned to an index (or key). In this case, the entry corresponds to the assigned bin. The component implementing this functionality receives an input index to select a destiny register, as well as a data word to be stored in the target register. Input is received through the *binData* bus. The target register is set using the *objIndex* port. The core component is a demultiplexer that routes the information to the desired register. It is also useful to query a register to retrieve its contents. This is achieved using the *rqObj* signal, used to control a multiplexer that is connected to all register outputs. The queried bin is then shown in the *rqBin* bus.

**Figure 14:**
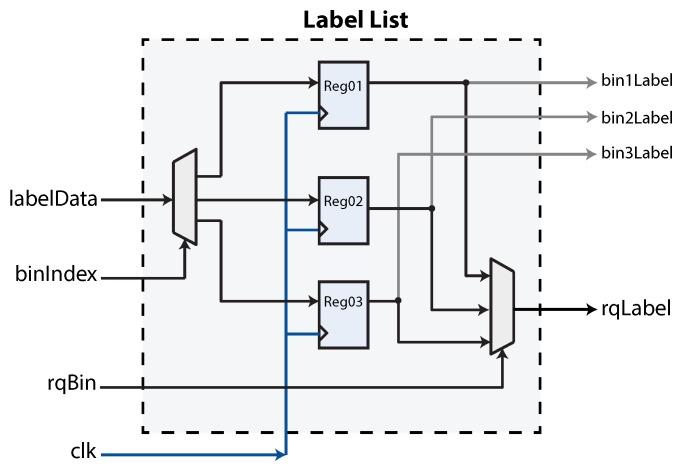
The label list implemented as a register-based array. In this figure, a maximum of three labels can be stored.

The label list hardware implementation is shown in Figure 14. This module is used to link a bin with a label. The idea and implementation is identical to the bin list. As before, a target bin is selected via the *binIndex* port, while a label is fed through the *labelData* signal. We have also included the same mechanism to query a label based on an input bin. This is accomplished with the *rqBin* signal. Lastly, the free bins list is also implemented as a register-based array. The free bins component includes an additional mechanism designed to show the latest free bin based on bin availability.

**Figure 15:**
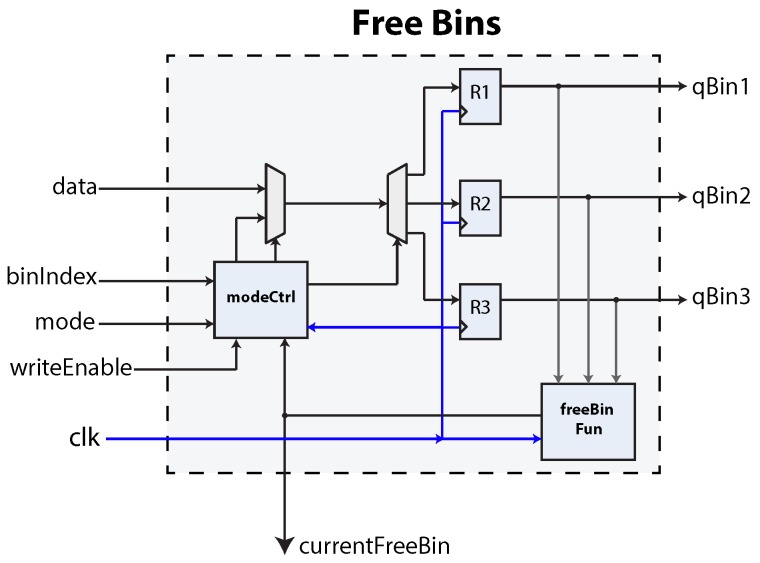
The free bins component. In this figure, a maximum of three bins can be used.

The hardware implementation is depicted in Figure 15. Input is received through the data bus. A bit is used to denote the status of a bin; an empty bin is marked with a logical zero, while a used bin is marked with a logical one. The target bin is set with the *binIndex* signal. Every time a bin is used, the input port must be filled with valid data, and the *writeEnable* signal must be asserted. This component features two operational modes: *manual* and *automatic*; set through the *mode* port.

Under manual operation, the control FSM must feed a target bin and a bit to mark it used or empty. Under automatic mode, the component will show the next available bin in the *currenFreeBin* port and will automatically mark it *used* as soon as *writeMode* is set. This mode helps to hasten bin assignation from the control FSM. The logic necessary to implement this functionality is stored in the *freeBin Fun* block. Each bin’s individual status can also be observed through the $qBin_{n}$ output ports.

The blob detection co-processor is realized on FPGA hardware with a consumption of 665 logic elements. The nominal working frequency is 50 MHz, and the maximum frequency is 125 MHz. The general co-processor specifications are depicted in Table 1. FPGA device specifications are also included. The maximum static power consumption has been estimated using Altera’s Power Consumption Documentation for Cyclone III family devices. Performance has been measured in frames per second (FPS). FPS measurement was performed at the RTL level. We used Altera’s Quartus software and Mentor Graphics’ Modelsim to time and inspect each processing stage of the architecture while input images were being fed. Performance was also measured by counting the number of frames per second that were processed on the FPGA board.

**Figure 16:**
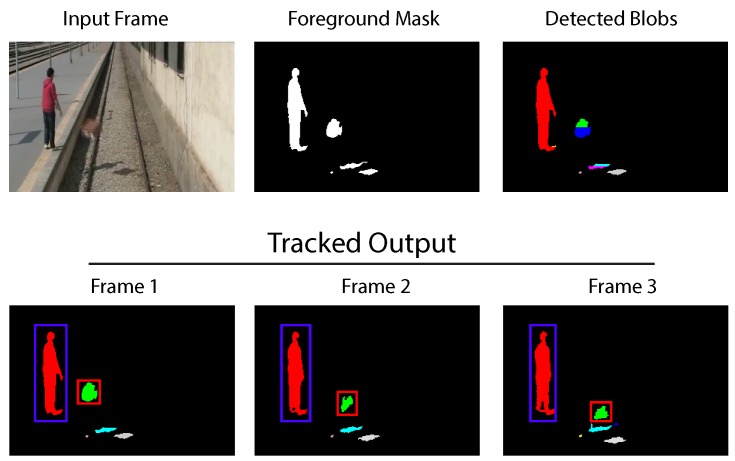
Results from the blob detection FPGA sub-system.

For test purposes, the system is tuned to track blobs based on their position and area only. Our application is interested in tracking the largest moving objects in a scene. Results obtained for this scenario are shown in Figure 16. A man throws a box at a rail-road in a desolate area. The scene is supervised with the automatic surveillance system focused on motion detection. The system’s output contains all of the detected blobs in the scene; a maximum number of SDO has been pre-set at ten blobs. After measurements are computed and correction is applied, the motion tracker identifies the target objects during three sequential frames. A total of eight labels is used, contained in ten bins.

**Figure 17:**
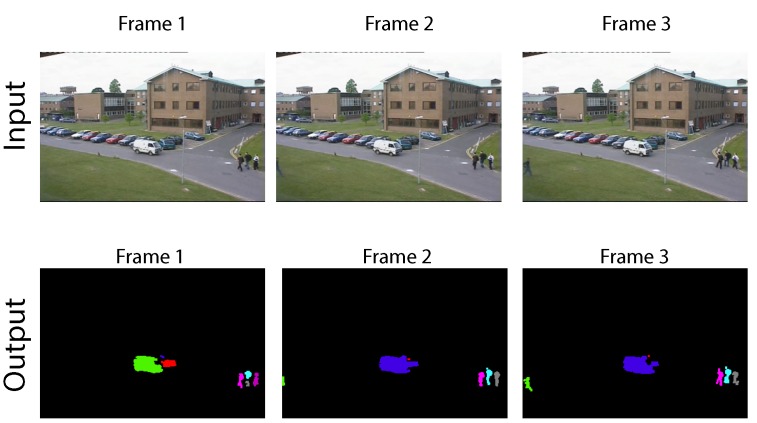
Outdoor PETS2001database blob detection.

Figure 17 presents a scene that has been taken from the performance evaluation of tracking and surveillance 2001 (PETS2001) database [66]. It features an outdoor surveillance view of a parking lot on a campus. No clean background of the scene is available; thus, the background modeling stage is trained with cars already parked. The figure depicts three input video frames and the blobs detected for each case, post-correction stage. There is moderate traffic of moving vehicles and persons, and the scene has been recorded under inconsistent light conditions, which causes some blobs to break into smaller pixel regions. Blobs also change labels between frames; however, our motion tracker detects this situation. A total of seven labels is used, contained in eight bins.

## 5. Results and Discussion

**Figure 18:**
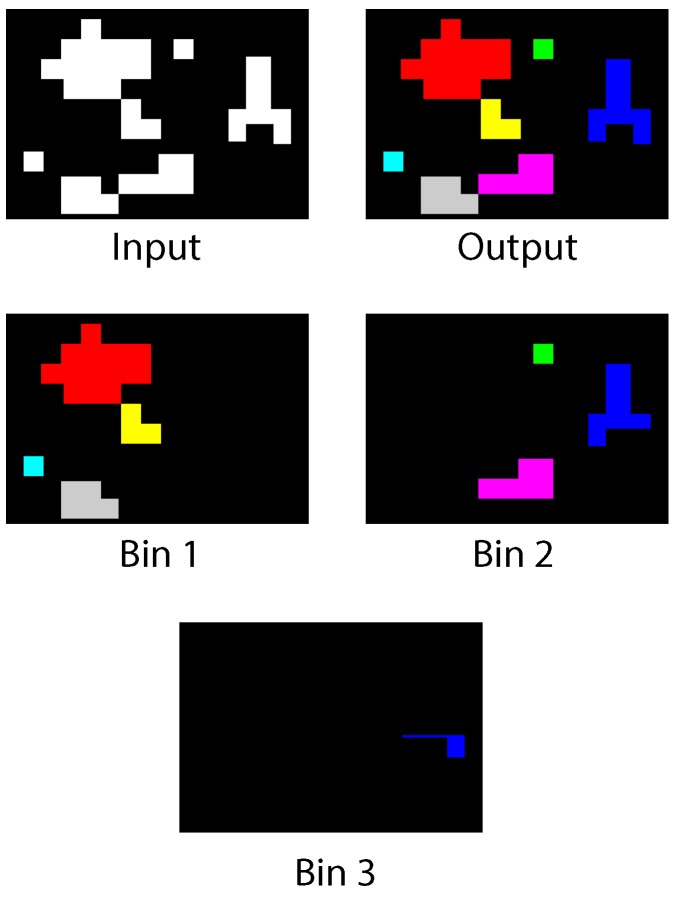
Test Image 1.

The blob detection algorithm is intended for general purpose recognition. As such, we evaluate its performance with various images that test general detection for use in other applications. On each test, the input image is scanned just once. The correction phase may be required for certain images after the first scan analysis is performed. If a common run in more than one bin is found, correction is applied by label merging. Figure 18 shows the first test image. The figure depicts the input binary mask, the blob detector output and the contents of each used bin. Blobs of different sizes and shapes are present, as well as multiple simultaneous disconnected blobs. For this image, a maximum number of three bins is used. As can be seen, a new bin is assigned every time a new SDO appears. As soon as a blob terminates and its data structures are freed, its original assigned bin can be used to store a new object. Bins 2 and 3 share a complex blob that cannot be stored using a single bin; however, its label is successfully shared across the two data structures.

**Figure 19:**
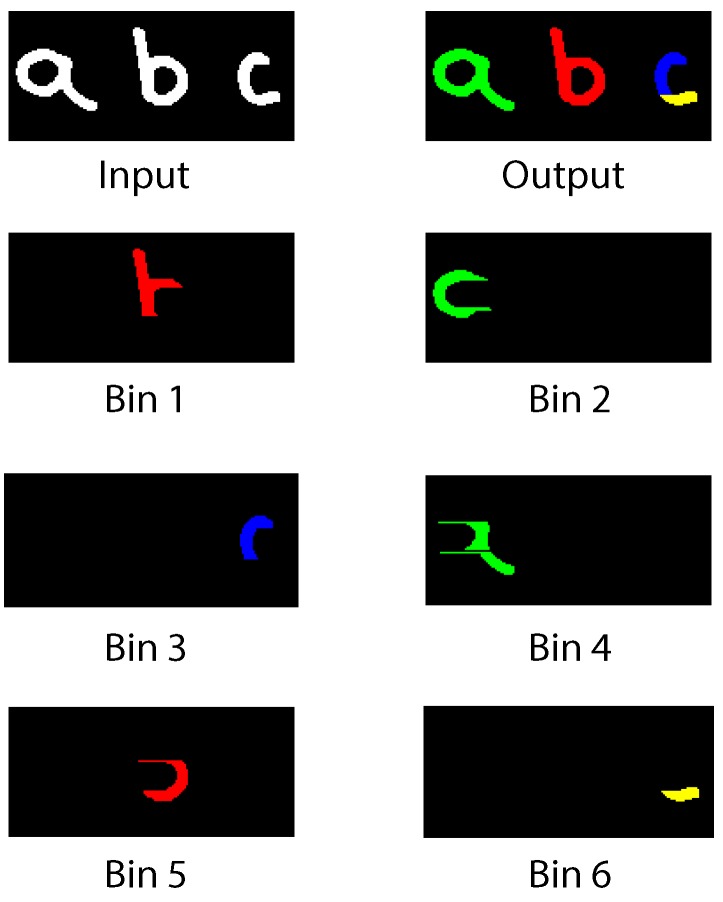
Test Image 2.

**Figure 20:**
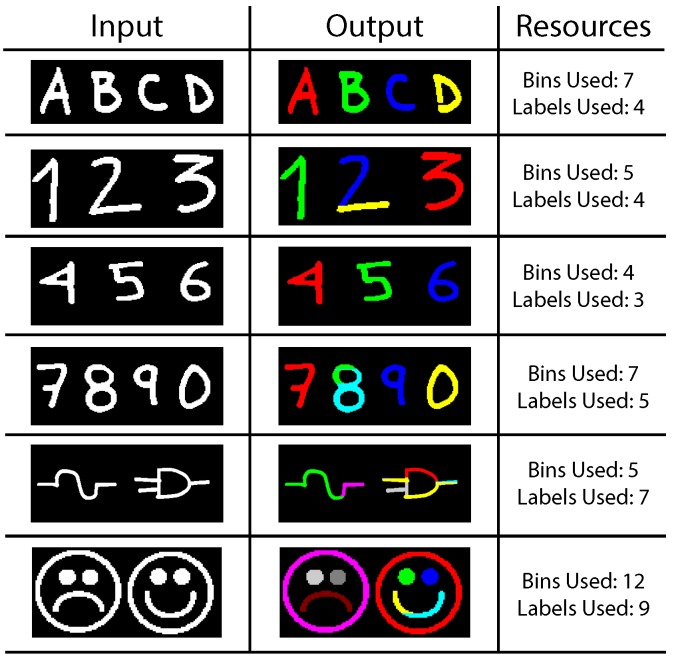
Complex test images.

Handwritten characters obtained from a sample of letters are used as test input in Figure 19. Three letters are tested simultaneously. Each character is stored in two bins. *a* and *b* are correctly assigned to a parent label across all their bins. A portion of the letter *c* is first identified as a separated blob; thus, a new (yellow) label is used instead of the parent (blue) label. Correction is applied on Bin 3 and Bin 6. Figure 20 shows an array of varied complex symbols. Only the input and output images are shown.

**Figure 21:**
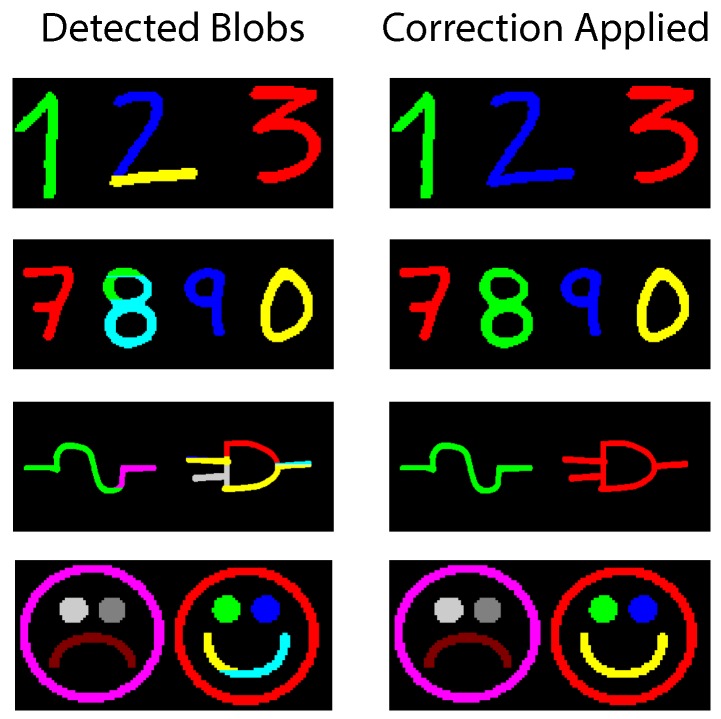
Complex test images after correction is applied.

**Figure 22:**
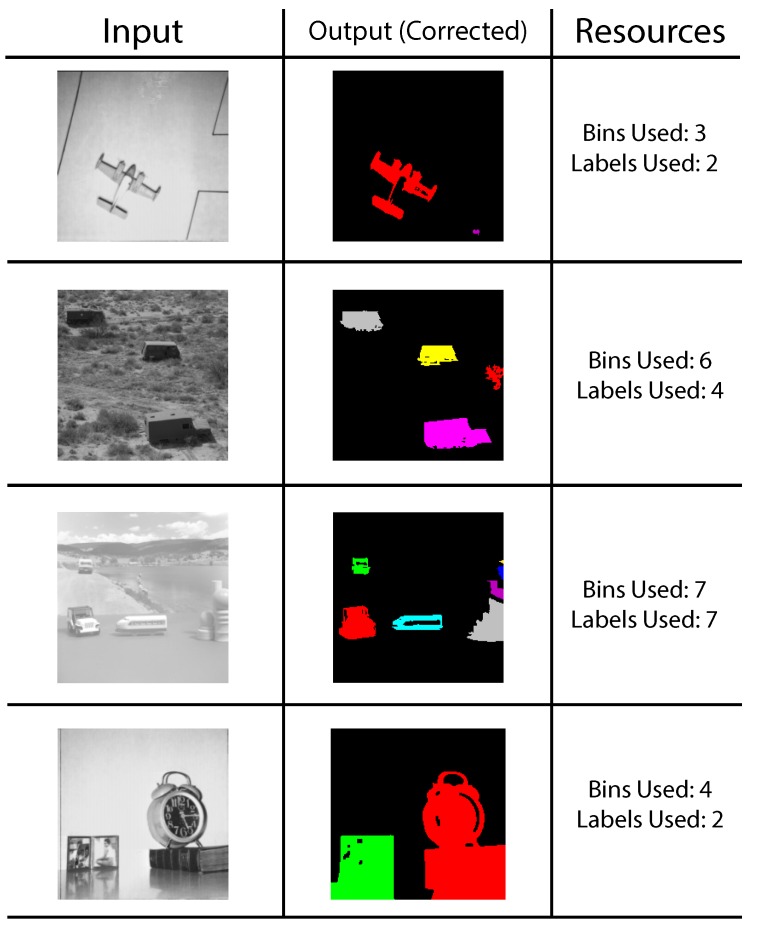
Input images from the USC-SIPI image database.

The resources needed to correctly detect each blob are also listed. For the most part, the test images are correctly identified without the need to apply the correction step. The two last images present a complicated mix of concave-down and concave-up shapes. Many of these shapes are not found to be joined until the last minute. The corrected results are depicted in Figure 21. Finally, input images from the University of Southern California Signal and Image Processing Institute (USC-SIPI) database [67] have also been tested. Most of these images show uniform blobs; however, big complex shapes are broken up into multiple bins; albeit, all sharing one single label. The frame size is of 200 × 200; the lack of detail in small images generally boosts performance. Results post-correction stage are shown in Figure 22.

**Figure 23:**
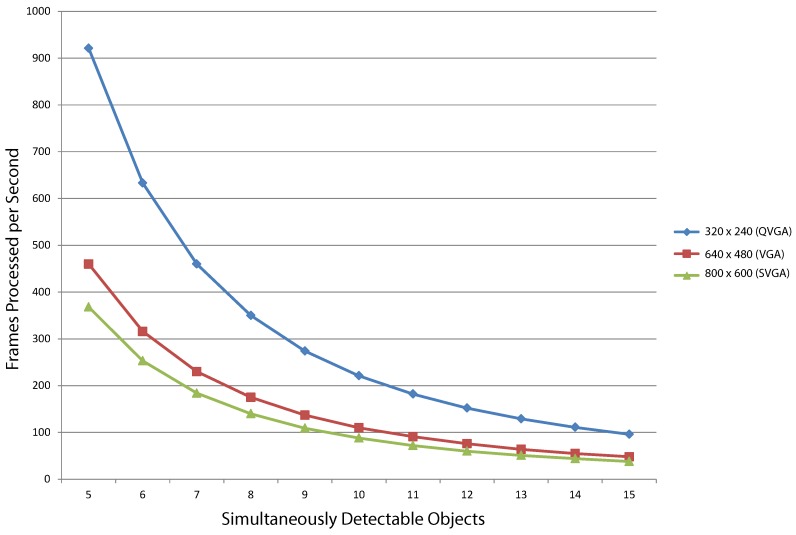
Simultaneously-detectable objects *vs.* frame processing rates for different image resolutions.

System latency depends directly on the number of simultaneously-detectable objects present in a single image row. Processing time increases quadratically to the input image’s size. Table 2 summarizes the maximum latency and frame processing rate consumed by three different image resolutions for a different number of maximum simultaneously detectable objects in one processing step. Performance for processing a full frame is also given. Similarly, Figure 23 depicts frame rates for maximum SDOs between five and 15. As mentioned in Section 3.6, computationally-heavy operations are carried out in the master control FSM. Classification of blob detection cases introduces the system’s main bottleneck. Nonetheless, processing frame rates are within real-time operating boundaries for most standard-definition applications. (e.g., 30 FPS). For this implementation test, processing has been fixed at a working system frequency of 50 MHz.

Table 3 summarizes the specifications of the FPGA-based implementations previously presented and compares them to the proposed architecture. Unfortunately, not all authors report all of their resource consumption parameters. Furthermore, the same frame size is not used across all architectures. We have included the bits of memory required for VGA frame buffering during the image acquisition stage. For an input QVGA image with one-bit data, pixels must be stored in a block of 76,800 (320 × 240) bits. Memory requirements for the rest of the resolutions are also shown. Our approach effectively minimizes memory consumption while meeting real-time requirements. For video surveillance applications, our work has found that a QVGA frame generally conveys enough detail for effective object and event detection. Some software solutions are presented in Table 4. Most achieve real-time performance; nevertheless, high-resource consumption makes embedded deployment difficult.

## 6. Conclusions

Blob detection is a common task for computer vision applications. It is often performed on general purpose computing architectures as an algorithm that relies on image storage. For implementation on embedded systems, however, system memory and computing power are limited resources, and alternate techniques must be designed. In this paper, a blob detection algorithm was proposed and developed for implementation on embedded hardware, focused on a system-on-chip application. Emphasis has been made on low-memory consumption and fast processing.

Blob information is stored in discrete data structures called bins. Bin management is achieved using linked-lists structures. It is important to note that blobs are dynamic objects with properties that can change within frames. Liked-lists keep track of these changes efficiently. Moreover, linked-lists present a good trade-off between design flexibility and resource consumption. Depending on blob dynamics, several cases that hinder optimal detection can be encountered. Complex shapes cannot be correctly identified on a single image scan and can lead to labeling errors. Such is the case of concave-up objects. To deal with this situation, an additional correction phase is also implemented. The correction phase only depends on the information contained in each data bin and does not require additional image scans or storage. Nonetheless, extra latency is added to the system.

Once a blob is detected, a label and a bin are assigned; blob data are processed for feature extraction, and a motion tracking stage can be used for blob seeking through multiple video frames. The system is used to track simple convex objects, composed of circular and squared contours. The correction phase has been seamlessly integrated in the detection module. A soft-core CPU is used to execute tracking software. The results obtained with this implementation show a fast blob detection of simple, but restricted shapes. The current version of the object sensing system is configured to detect a maximum number of ten blobs presented simultaneously (*i.e.*, in one single row of the input image).

We evaluated the performance of the blob detection algorithm implementing a full vision-based object detection system built on FPGA hardware. The application focuses on automated video surveillance. The system integrates background modeling and blob detection co-processors. A simple tracking algorithm based on the minimum distance vector is used and the results obtained while monitoring a fixed area shown. Tracking is intended to demonstrate the feasibility of our blob detection approach as a basis for higher level operations. Our system is capable of detecting basic motion based on blob position and size on a low traffic area in real time. The system is, however, currently locked to a detection of 10 simultaneously-detectable objects. Items that exceed such a threshold are simply not detected due to the absence of resources.

Results evaluating character recognition are also included. Character recognition features complex shapes; however, the algorithm shows an adequate detection rate in most of the cases. The precision of the algorithm is always restricted by the number of maximum simultaneously-detectable objects and the number of bins available. As the complexity of the detected objects increases, the resources needed to handle multiple object data also raises. The algorithm can be used in applications that require character recognition of small images composed of simple strings and custom symbols. More complex characters will need the definitions of new detection cases.

In this work, full image storage is traded for simplified description data stored using linked-lists. Although the proposed hardware implementation focuses on QVGA (320 × 240) resolutions, we present two additional variants that process VGA (640 × 480) and SVGA (800 × 600) frame sizes. Binary input can be obtained directly from the acquisition stage (e.g., a black and white sensor) or computed by an additional processing module (e.g., background pixel classifier). In either case, the binary image must be first stored in memory to be read by our blob detection module. Processing latency grows quadratically to the frame size. However, real-time constraints are met for all of our target resolutions.

## Figures and Tables

**Table 1:** Blob detection architecture specification.

Parameter	Value
FPGA Technology	Altera Cyclone III
FPGA Chip Used	EP3C120
Max. Frequency	125 MHz @ $100{\;}^{\circ }$C
Min. Frequency	112.74 MHz @ $-40{\;}^{\circ }$C
Working Frequency	50 MHz
Input Image Width	320 px
Input Image Height	240 px
Input Pixel Depth	1 bit
Processing Time per Frame	4.52 ms
Processed Frames Per Second	221 FPS
Logic Elements Consumption	665
Max. Static Power Consumption	0.17 Watts

**Table 2:** Blob detection latency and frame processing rate.

	Latency Per		Frame Performance		
	Processing Stage (50 MHz)		320 × 240	640 × 480	800 × 600
Objects	Clock Cycles	Seconds	(FPS)	(FPS)	(FPS)
1	14	$6.7\times 10^{-10}$	14,880	7440	5952
2	37	$1.8\times 10^{-4}$	5630	2815	2252
3	80	$3.8\times 10^{-4}$	2604	1302	1041
4	143	$6.9\times 10^{-4}$	1456	728	582
5	226	$1.1\times 10^{-3}$	921	460	368
6	329	$1.6\times 10^{-3}$	633	316	253
7	452	$2.2\times 10^{-3}$	460	230	184
8	595	$2.9\times 10^{-3}$	350	175	140
9	758	$3.6\times 10^{-3}$	274	137	109
10	941	$4.5\times 10^{-3}$	221	110	88

**Table 3:** Comparison of blob detection FPGA architectures.

Hardware	Platform	Frame	Pixel	Memory	Frequency	FPS
Architecture		Size	Depth	Usage		
Calvo-Galle *et al.*	Xilinx Spartan-3A	640 × 480	8 bit	2, 462, 720 bits	27 MHz	60
Kiran *et al.* (1)	Xilinx Virtex V	100 × 100	8 bit	144, 000 bits	100 MHz	4545
Kiran *et al.* (2)	Xilinx Virtex V	1024 × 1024	8 bit	N/A	100 MHz	61
Mauch *et al.*	Xilinx Spartan-6	224 × 224	12/10 bit	162,000 bits	70 MHz	905
Klaiber *et al.* (2013)	Xilinx Virtex VI	1024 × 1024	N/A	1,512,000 bits	136.4 MHz	1049
Klaiber *et al.* (2015)	Xilinx Kintex 7	7680 × 4330	N/A	548,000 bits	170.3 MHz	N/A
Yuhabi *et al.*	Altera Stratix II	1280 × 1280	1 bit	75,600 bits	97.4 MHz	49
Bochem *et al.*	Altera DE2 Cyclone II	640 × 480	N/A	239, 316 bits	125 MHz	50
**Proposed**						
QVGA	Altera Cyclone III	320 × 240	1 bit	76,800 bits	50 MHz	221
VGA	Altera Cyclone III	640 × 480	1 bit	307,200 bits	50 MHz	110
SVGA	Altera Cyclone III	800 × 600	1 bit	480,000 bits	50 MHz	88

**Table 4:** Comparison of blob detection software algorithms.

Algorithm	Technology	Frame	Memory	Processing	FPS
		Size		Time	
Paralic *et al.* (1)	Intel Atom N280	320 × 240	1 GB	1.13 ms	30
Paralic *et al.* (2)	Intel Atom N280	640 × 480	1 GB	4.86 ms	30
Binh *et al.*	Pentium 4 (3 GHz)	320 × 240	3 GB	3.72 ms	N/A
Swati *et al.*	Intel Core 2 Duo	800 × 1200	3 GB	21 ms	N/A
Oro *et al.*	NVidia GTX 470 GPU	1920 × 1080	1.2 GB	2.3 ms	35
Kumar *et al.*	TriMedia DSP	640 × 480	N/A	4.2 ms	30
